# Strain-specific Fibril Propagation by an Aβ Dodecamer

**DOI:** 10.1038/srep40787

**Published:** 2017-01-18

**Authors:** Dexter N. Dean, Pradipta K. Das, Pratip Rana, Franklin Burg, Yona Levites, Sarah E. Morgan, Preetam Ghosh, Vijayaraghavan Rangachari

**Affiliations:** 1 Department of Chemistry and Biochemistry, University of Southern Mississippi, Hattiesburg, MS 39406, USA; 2 School of Polymers and High Performance Materials, University of Southern Mississippi, Hattiesburg, MS 39406, USA; 3 Department of Computer Science, Virginia Commonwealth University, Richmond, VA 23284, USA; 4 Center for Translational Research in Neurodegenerative Disease, University of Florida, Gainesville, FL 32611, USA.

## Abstract

Low molecular weight oligomers of amyloid-β (Aβ) have emerged as the primary toxic agents in the etiology of Alzheimer disease (AD). Polymorphism observed within the aggregation end products of fibrils are known to arise due to microstructural differences among the oligomers. Diversity in aggregate morphology correlates with the differences in AD, cementing the idea that conformational strains of oligomers could be significant in phenotypic outcomes. Therefore, it is imperative to determine the ability of strains to faithfully propagate their structure. Here we report fibril propagation of an Aβ42 dodecamer called large fatty acid-derived oligomers (LFAOs). The LFAO oligomeric strain selectively induces acute cerebral amyloid angiopathy (CAA) in neonatally-injected transgenic CRND8 mice. Propagation *in-vitro* occurs as a three-step process involving the association of LFAO units. LFAO-seeded fibrils possess distinct morphology made of repeating LFAO units that could be regenerated upon sonication. Overall, these data bring forth an important mechanistic perspective into strain-specific propagation of oligomers that has remained elusive thus far.

Alzheimer disease (AD) is a neurodegenerative disorder that results in acute memory loss and cognitive decline in patients over the age of 60. AD is one of several pathological conditions that fall within the increasing pool of protein misfolding disorders, including Parkinson disease, Huntington disease, amyotrophic lateral sclerosis and Creutzfeldt-Jakob disease (CJD). The common underlying mechanism observed in many neurodegenerative disorders is the self-assembly of proteins towards pathogenic aggregates called ‘amyloids’, one that leads to a gain of function toxicity. In the case of AD, the polypeptide amyloid-β (Aβ), generated by the cleavage of amyloid precursor protein, undergoes aggregation to form insoluble fibers often clustered as senile plaques in brains of AD patients^1^. The commonality in the mechanism of aggregation among many amyloid proteins has raised the question of whether these diseases may also share other structural and functional behaviors^2^,^3^. One property of interest is the transmissibility observed in prion diseases, which has gathered increased attention lately^4^. In prion diseases, the pathogenic strain (PrP^Sc^) is faithfully propagated without structural distortions in a template-assisted mechanism^5^. Phenotypic diversity in CJD, such as distinct neuropathological patterns, can be attributed to specific PrP^Sc^ strains^6^. Seeding of Aβ fibril formation is also a template-assisted process, and micro-structural heterogeneity in the seed results in diverse strains of fibrils depending on their growth conditions^7^,^8^. Although transmissibility of Aβ aggregates in AD is far from clear, increasing evidence support a prion-like propagation of Aβ *in vivo*^9^,^10^.

In light of the emerging possibility that phenotypic diversity in AD arises from the heterogeneity among Aβ aggregates, it is imperative to investigate these mechanisms in detail. Particularly unclear in such a mechanism is the role of conformationally unique low molecular weight (LMW) oligomers. We have previously established that an Aβ dodecamer, called large fatty acid-derived oligomers (LFAOs), undergoes amplification by a templated process^11^,^12^,^13^,^14^. LFAOs, generated in the presence of fatty acid interfaces, are formed along an alternative pathway (off-pathway) to be termed a conformational ‘strain’. Due to its increased half-life, LFAOs involve in fibril-free, monomer-oligomer/oligomer-oligomer interactions towards lateral propagation of oligomer seeds^12^. Recent insights into their mechanisms revealed that unlike LFAO 12mers, dimeric LFAOs (disperse 12-24mers) resist replication^14^(Fig. 1, reproduced from our work^14^). In the current report, we examine the ability and mechanism of high concentrations of LFAOs (12-24mers) to undergo prototypical prion-like propagation towards LFAO strain-specific fibrils. Our results indicate that only the LFAO (12-24mer)-seeded aggregates, and not unseeded or fibril-seeded aggregates, form a propagating unit (propagon) en route to the formation of morphologically-distinct fibrils. Together, in conjunction with our previous report^14^, the current results reveal a novel three-stage mechanism of an Aβ oligomer (LFAOs) involving the sequential generation of a replicating oligomer (replicon), a propagating unit (propagon), and fibrils.

## Results

### LFAOs selectively induce widespread amyloid deposition and CAA in TgCRND8 mice

Based on our biophysical and cellular characterization of LFAOs^11^,^12^,^13^,^14^, as well as the previously observed prion-like propagation of amyloid seeds in transgenic animals^3^, we hypothesized that introduction of LFAOs to the neonatal brain of an AD transgenic mice will result in propagation of oligomers, and widespread pathology in brains. To test this hypothesis, buffered Aβ monomers, LFAOs and fibrils (10 μM) along with a PBS control were injected into the cerebral ventricles of three litters of newborn TgCRND8 mice. It has been established that the administration of α-synuclein (αS) aggregates into the brains of neonatal mice catalyzes the spread of intracellular αS pathology^15^. TgCRND8 mice were chosen for our experiments as they overexpress human APP with KM670/671NL (Swedish) and V717F (Indiana) mutations at levels approximately 5-fold higher than endogenous murine APP. This leads to significant deposition of amyloid plaques within three month of age^16^. After three months, the mice were euthanized and brains were extracted. One hemibrain was frozen for further biochemical analysis, while the other was fixed in 4% paraformaldehyde, paraffin embedded and processed for immunostaining. Upon analysis, the control mice injected with PBS showed a sparse number of small amyloid deposits both in the cortex and hippocampus (Fig. 2a). Aβ LFAO and fibril administration resulted in an increased number of amyloid plaques throughout the cortex and hippocampus as well as diffuse deposits along the anatomical structure of the hippocampus and the performant path (Fig. 2a and d). Amyloid burden in mice injected with Aβ fibrils was the highest of all groups followed by LFAO injected mice (Fig. 2b). However, interestingly, LFAO-injected mice developed profound CAA, which results from the accumulation of amyloid in blood vessels, with a large number of Aβ positive vessels in the meninges and choroid observed (Fig. 2a and c). This suggests that not only LFAOs are capable of inducing widespread pathology, they are also is selective to induce CAA as opposed to or unseeded monomers. Furthermore, biochemical analysis were performed on the frozen hemibrains by sequential fractionation in radioimmunoprecipitation assay (RIPA), SDS 2% and formic acid 70%, and Aβ levels were measured by sandwich ELISA. All Aβ species caused an increase in both Aβ40 and Aβ42 levels in SDS and FA fractions (there is no change in RIPA soluble Aβ levels, data not shown). The increase is the highest in the brains of mice injected with fibril seeds. Overall, we demonstrate a 3–4-fold increase in Aβ40 and Aβ42 in SDS, and a 7–8 fold increase in FA fractions, following neonatal administration of fibril and LFAO seeds. Together, these suggest that LFAOs are capable of inducing propagation and widespread deposition of amyloids, and are selective in inducing CAA, to fibrils in Tg mice brains.

### LFAO-seeded Aβ aggregates display distinct biophysical characteristics

**Figure 1:**
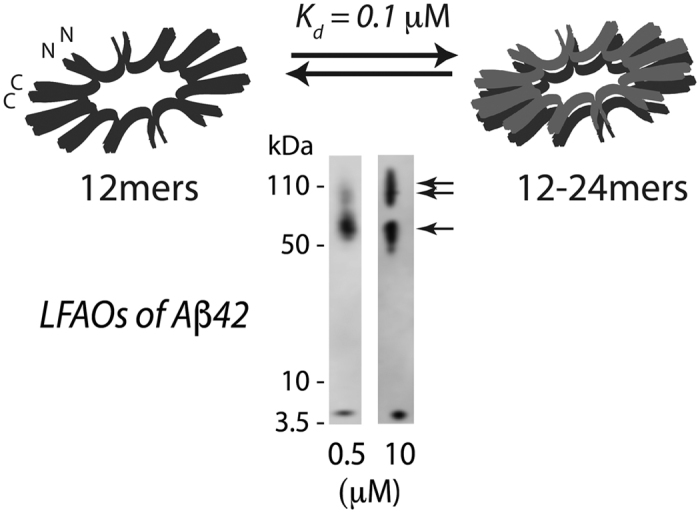
Conformational dynamics of Aβ42 LFAO dodecamers. LFAOs have been previously shown to undergo a concentration-dependent transition between discrete 12mers (56 kDa, single arrow) to disperse 12-24mers (110 kDa, double arrow) with a dissociation constant (K_d_) of 0.1 μM. Reproduced with permission^14^.

**Figure 2:**
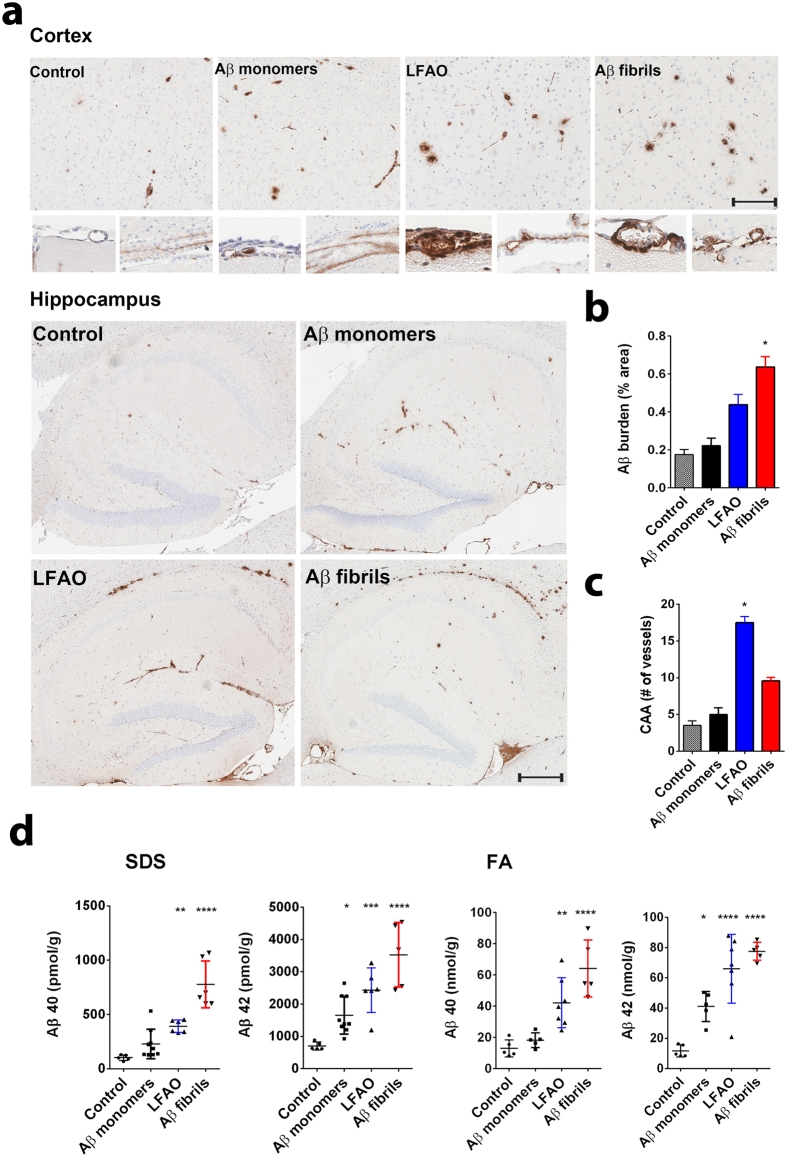
LFAOs selectively induce widespread amyloid deposition and CAA in TgCRND8 mice. (**a**) Newborn CRND8 mice were injected with 4 μL Aβ fibrils, LFAO, Aβ monomers (10 μM) in the cerebral ventricles. Control group received PBS. 3 months later brains were extracted and one hemibrain fixed and stained with anti-pan- Aβ mAb5Biotin antibody. Amyloid staining of plaques and CAA in the representative paraffin sections is shown in the cortex, meningeal vessels and choroid plexus and in the hippocampus of injected mice. Scale Bar, 500 μm (cortex and CAA), 250 μm (hippocampus). (**b**) Quantification of Aβ positive immunostaining shows significantly increased amyloid plaque burden (immunostained with anti-pan- Aβ mAb5Biotin) Aβ fibrils injected mice compared to control mice. Data represents mean ± sem. n = 6–10/group. ***p < 0.01, unpaired two-tailed t test. (**c**) Quantification of CAA. Aβ positive positive blood vessels in the meningies and throughout the brain tissue were evaluated in a blind manner and given a qualitative score from 0 to 3. Vessels with scores 2 or 3 were counted. LFAO injected mice have significantly higher levels of CAA compared to control. Data represents mean ± sem. n = 6–10/group. ***p < 0.01, unpaired two-tailed t test. (**d**) Biochemical analyses of sequentially extracted Aβ42 and Aβ40 levels by end-specific sandwich ELISA show significantly increased SDS soluble and formic acid extractable insoluble Aβ levels in LFAO and Aβ fibrils injected mice compared to control mice. Small increase in Aβ levels was also detected in Aβ monomer injected mice. Data represents mean ± sem. n = 6–10 mice/group. N = 6–10, *p < 0.05, **p < 0.01, ***p < 0.001, ****p < 0.0001, 2 way Anova with Tukey’s multiple comparison test).

**Figure 3:**
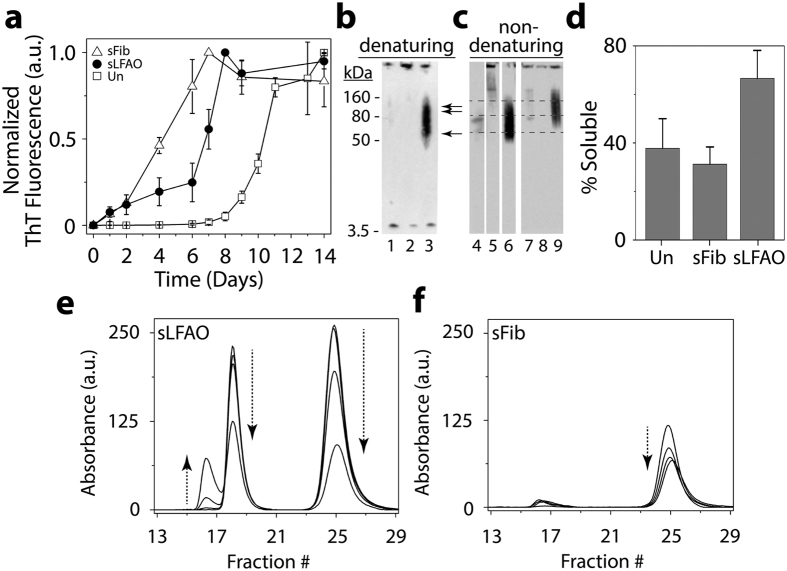
LFAO-seeded Aβ aggregates display distinct biophysical characteristics. (**a**) ThT fluorescence aggregation kinetics of Aβ monomer (50 μM) in the absence of seed (Un, □) or seeded with 10 μM LFAOs (sLFAO, ●) or fibrils (sFib, △). (**b**) Denaturing (SDS) PAGE of Un (1), sFib (2), and sLFAO (3) at 14 days of incubation. The single and double arrows represent LFAO 12 (56 kDa) and 24mer (110 kDa), respectively. (**c**) Non-denaturing (native) PAGE of parent monomer (4), fibril (5), and LFAO (6) along with Un (7), sFib (8), and sLFAO (9). The lower, middle, and top dashed lines represent native migration of LFAO 12mer, 24mer, and HMW oligomeric species, respectively. 540 ng was loaded into each well for all samples. (**d**) Percentage of solubility, as determined by centrifugation and ThT fluorescence, at 28 days of incubation (14 days at 25 °C, 14 days at 4 °C). (**e**–**f**) Quantitative SEC of sLFAO (**e**) and sFib (**f**) at 4, 5, 6, and 7 days of incubation, respectively.

In our previous report, we established that LFAOs undergo a transition from 12mers to more disperse 12-24mers in a concentration-dependent manner (Fig. 1, reproduced from^14^). We also identified that 12mers undergo replication preferentially over 12-24mers. However, characteristics of 12-24mer LFAOs, in particular their ability to faithfully propagate towards a distinct fibril strain, was unclear, although they propagate within mice brains (Fig. 2). Therefore, to further investigate the behavior of 12-24mer LFAOs, freshly purified Aβ monomer (50 μM) was seeded with 10 μM LFAO seeds, a concentration at which LFAOs are predominantly 12-24mers^14^, and the aggregation was monitored by thioflavin-T (ThT) fluorescence. As a positive control, Aβ monomer was seeded with fibril seeds, while Aβ monomer in the absence of a seed was used as a negative control. ThT signal artifacts, if any, were eliminated from the data by appropriate negative controls such that the intensity increase solely reflected emerging amyloid aggregates. The unseeded Aβ control (Un) showed a typical sigmoidal growth pattern with a lag time of 8 days (Fig. 3a, □). As expected, monomers seeded with 10 μM fibril seeds (sFib) did not show an observable lag time and resulted in rapid growth towards fibrils (Fig. 3a, △). However, monomers seeded with LFAOs (sLFAO) displayed a distinct two-phase growth curve (Fig. 3a, ●). A slow exponential increase during the first six days of incubation followed by rapid increase in ThT fluorescence and saturation during the next eight days was observed (Fig. 3a, ●). Based on ThT profiles alone, it is clear that LFAO-seeded aggregation behaves differently from the fibril-seeded control. To analyze whether the differences in sLFAO and sFib aggregation is manifested in the nature of fibrils formed, the samples were electrophoresed and immunoblotted after 14 days of incubation (Fig. 3b,c). Under denaturing conditions (SDS-PAGE), both Un and sFib showed the presence of high molecular weight (HMW) aggregates that did not enter the gel, indicative of fibrils (Fig. 3b, lanes 1–2). However for sLFAO, in addition to the HMW bands, a significant band corresponding to 12-24mers (56–110 kDa) was observed (Fig. 3b, lane 3). The faint monomeric (~4.5 kDa) band observed in all three samples could correspond to either unreacted monomers or those that are generated upon denaturation of HMW species by SDS. To obtain more clarity on the banding patterns observed, the samples were also electrophoresed under non-denaturing conditions (Fig. 3c). While monomeric Aβ showed a narrow single band (Fig. 3c, lane 4), fibril seed (lane 5) and parent LFAOs (pLFAOs; lane 6) showed a HMW band that failed to enter the gel and a broad band, respectively. As expected, both Un and sFib samples showed HMW bands after 14 days of incubation (lanes 7 and 8) while sLFAO showed the presence of bands corresponding to LMW and HMW species (lane 9). This experiment confirms two things: *i*) the monomeric bands observed in denaturing gels are likely due to the dissociation of aggregates in 1% SDS contained in the sample buffer, and *ii*) sLFAOs contained a significant amount of soluble oligomers even after 14 days of incubation as opposed to sFib and Un samples, which could indicate a greater degree of fibril fragmentation in the sLFAO sample. Furthermore, distinct differences in migration patterns were observed between pLFAOs and sLFAOs in the native gel, which may also suggest that oligomers observed after 14 days are larger than 12-24mers. To further probe the percentage of soluble aggregates present in each sample, the samples were centrifuged after a total of four weeks of incubation (14 days at 25 °C + 14 days at 4 °C) at 18,000 × g for 20 min, and the supernatants were analyzed for ThT fluorescence (Fig. 3d). Both Un and sFib showed 30–40% of soluble aggregates while sLFAO showed ~65% of aggregates that did not spin down upon centrifugation (Fig. 3d).

The biphasic nature of LFAO seeded aggregation could indicate multiple phase transitions during aggregation, and thus may involve intermediates that are stable enough to be isolated. Therefore, in order to better understand the transitions and characteristics of LFAO seeded aggregation, size exclusion chromatography (SEC) was utilized. Aliquots of samples from the reaction in Fig. 3a were taken periodically for SEC analysis at 4, 5, 6, and 7 days of incubation. After four days of incubation, sLFAO sample fractionated to two predominant peaks, one at fraction 18 and another at fraction 25 (monomer) (Fig. 3e). As the reaction progressed, a third peak eluting near the void volume (fraction 16) increased as the more inclusive peaks decreased, respectively. This indicates that the inflection point in sLFAO ThT response at 5–7 days corresponds to a stable, isolable species. Fractionation of sFib samples on the other hand showed only two species during the same time, with the absence of more inclusive peak at fraction 19 (Fig. 3f). The peak eluting near the void volume (fraction 16) however, did not increase with time as observed for sLFAO sample. This is mainly due to majority of the fibrils being removed by centrifugation prior to fractionation (Fig. 3d). AFM analysis revealed the sLFAO peak at fraction 19 corresponds primarily to two species; spherical particles corresponding to parent LFAOs^12^, and small elongated fibrils corresponding to an intermediate species (Supplementary Fig. 1a). AFM also revealed that the peak at fraction 16 to be larger fibrils of 1–2 μm in length (Supplementary Fig. 1b). The peak eluting at fraction 25 corresponded to monomeric Aβ. The phase transitions in sLFAO were further investigated in detail by numerical simulation methods and are discussed later in the manuscript. Together the data reveal that LFAO-seeded aggregation behaves differently from the fibril-seeded control (Fig. 3f). Taken together, the data reveal that LFAO-seeded aggregates are more amorphous than those generated in the absence of LFAO seeds, and may possess distinct morphological and biophysical properties.

### sLFAO fibrils show unique, ‘repeat morphology’

**Figure 4:**
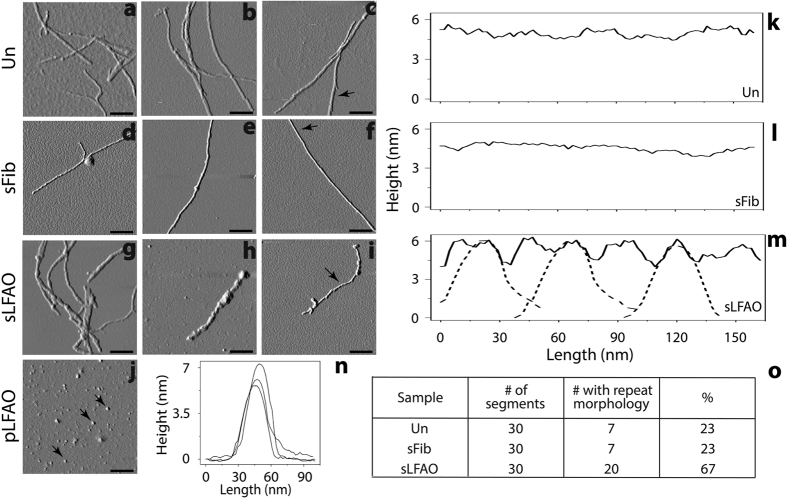
sLFAO fibrils reveal unique repeat morphology. (**a–j**) AFM images of Un (**a–c**), sFib (**d–f**), and sLFAO (**g–i**) aggregates at 10 days of incubation, along with pLFAOs (**j**). Scale bars represent 200 nm. (**k–n**) Surface morphology analysis of Un (**k**), sFib (**l**), sLFAO (**m**, solid), and pLFAOs (**n**), as indicated by black arrows. The dashed lines in panel **m** represent data from pLFAOs in panel **n**. (**o**) Statistical analysis of AFM images, as described in the text.

To see whether sLFAO samples indeed generate fibrils with distinct morphology, the samples were visualized by atomic force microscopy (AFM) after 10 days of incubation. Both Un (Fig. 4a–c) and sFib (Fig. 4d–f) aggregates showed long, smooth fibrils with an average cross-sectional height of 6.3 ± 0.45 and 6.1 ± 0.84 nm, respectively. sLFAO aggregates on the other hand displayed a slight decrease in the average cross-sectional height with 5.8 ± 0.36 nm (Fig. 4g–i). Although heights were relatively similar among all the samples, further detailed analysis provided insights into the aggregate characteristics. Analyses of the surface morphology along the length of the fibrils revealed an overall smooth morphology for Un (Fig. 4k) and sFib (Fig. 4l) aggregates. However, sLFAO aggregates showed a feature that appears to repeat itself along the fibril axis (Fig. 4m). Such a repeat morphology was clearly absent in both Un and sFib samples. AFM of pLFAO seeds show punctate spherical particles with an average height of 6.3 ± 0.87 nm (Fig. 4j and n). This height matches the individual ‘peaks’ observed in sLFAO repeat morphology (Fig. 4m, dashed line), supporting the contention that sLFAO aggregates contain repeating LFAO units. In order to ascertain that the repeat morphology feature was statistically significant, multiple images at various fields were analyzed. For every sample, a total of 30 individual 150 nm length segments were analyzed. A pattern was considered to be a ‘repeat morphology’ if two or more contiguous peaks and valleys with a change in height of 1.5 nm or more were observed. Upon analyzing the images using these parameters, sLFAO aggregates contained 67% of a repeat morphology, while only 23% was observed for both Un and sFib (Fig. 4o), providing a statistical insight to the unique morphology formed by sLFAO aggregates.

### Propagating units of sLFAO fibrils can be generated upon sonication

**Figure 5:**
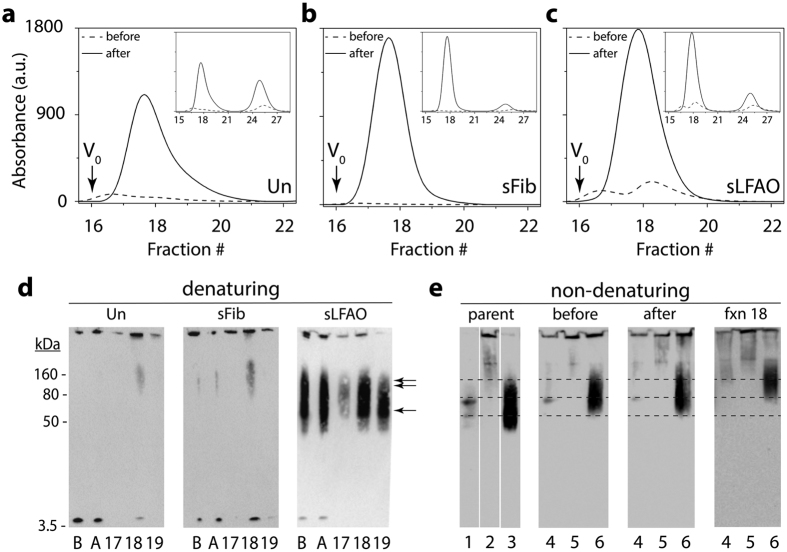
Generation of smaller propagating units of sLFAOs called iLFAOs. (**a–c**) SEC chromatograms before (dashed) and after (solid) sonication for Un, sFib, and sLFAO, respectively. The downward arrow represents the void volume (V_0_) of the column. *Inset*) Chromatograms showing the elution of oligomeric fragments and monomers. (**d**) Immunoblot of aggregates under denaturing conditions before (B) and after (A) sonication as well as fractions (17–19) from SEC after sonication. Single and double arrows represent LFAO 12 and 24mer, respectively. (**e**) Non-denaturing (native) immunoblot of parent monomer (1), fibril (2), and LFAO (3), along with Un (4), sFib (5), and sLFAO (6) samples before and after sonication, as well as fraction 18 from fractionation after sonication. The lower, middle, and top dashed lines represent the native migration of LFAO 12mer, 24mer, and HMW oligomeric species. 540 ng was loaded into each well for all samples.

If LFAOs propagate as distinct individual units, a question arises as to whether it is possible to re-generate smaller propagating pLFAO units by breaking down sLFAO aggregates. Generation of such ‘seeding ends’ has been well established for PrP aggregates using the protein misfolding cyclic amplification (PMCA) assay, in which aggregates are subjected to repeated cycles of sonication and incubation^17^. Therefore, to see whether sonication of Un, sFib and sLFAO fibrils could regenerate the propagating units, the samples were sonicated using 80% power level (Fig. 5). After 28 days of incubation, aliquots of samples before and after sonication were analyzed using SEC. Fractionation of all samples after sonication resulted in predominantly two fractions, one that eluted near the void volume (V_0_; fractions 17–19) corresponding to the oligomeric aggregates, and another at more inclusive volume corresponding to monomer (fractions 24–27) (Fig. 5a–c). For all the samples, the elution volumes of the oligomeric peak were slightly more inclusive than those prior to sonication, suggestive of reduction in aggregate size (Fig. 5a–c). Sonication of sFib (Fig. 5b) and sLFAO (Fig. 5c) resulted in approximately similar amounts of fragmented aggregates as opposed to Un (Fig. 5a), which had an approximately ~60% decrease. Furthermore, the Un sample also showed the maximal increase in the fractions corresponding to monomers after sonication (Fig. 5a, inset), suggesting that sonication not only generated smaller aggregates but also significant amount of monomers in Un samples. The increase in monomer amounts also corresponds to the decreased amounts of Un aggregate fragments generated. The sFib sample showed the least degree of monomer generation upon sonication (Fig. 5b, inset). Immunoblotting of these samples in parallel under denaturing conditions (Fig. 5d) showed an oligomeric species at ~160 kDa in fraction 18 of Un and sFib as a result of sonication or dissociation of a larger fragment in denaturing conditions (1% SDS). Similarly, the monomeric band observed in these samples could also result from the dissociation of the aggregate fragments during electrophoresis. The sLFAO sample on the other hand, showed the presence of substantial amount of oligomers corresponding to disperse 12-24mers similar to the pLFAOs in fraction 18 (Fig. 5d). Immunoblotting under non-denaturing conditions revealed that all three samples generated oligomers in addition to monomers during sonication (Fig. 5e). sLFAOs under non-denaturing immunoblotting (fraction 18) showed more intense oligomer bands than Un and sFib samples (Fig. 5e). Importantly, the oligomer bands observed in sLFAO (Fig. 5e, lane 6) correspond to a slightly higher molecular weight to that of pLFAOs (Fig. 5e, lane 3) and those observed in the denaturing immunoblot (Fig. 5d). This suggests that what appeared to be the presence of pLFAOs (12-24mers) under denaturing conditions (Fig. 5d) are likely due to the dissociation of larger oligomers by SDS treatment. Collectively, the data suggest that sLFAO fibrils are susceptible to fragmentation by sonication, and generate smaller fragments (from here on called isolated-LFAOs; iLFAOs), which can be isolated. However, it is also clear that sonication of sLFAO fibrils does not directly result in the generation of pLFAO seeds (12-24mers) but rather larger, stable, iLFAOs (iLFAOs ≠ pLFAOs) that manifests in the biphasic aggregation profile observed (Fig. 3a).

### iLFAOs appear to form by the association of multiple pLFAO units

**Figure 6:**
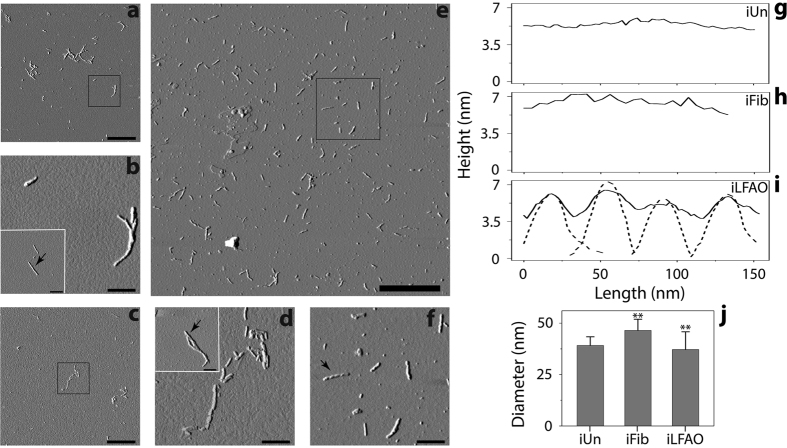
Unique repeat morphology is retained within iLFAOs. (**a–f**) AFM images of sonicated and isolated (fraction 18) Un (iUn, **a**,**b**), sFib (iFib, **c**,**d**), and sLFAO (iLFAO, **e**,**f**). *Inset*) AFM images used for surface analysis. Scale bars for (**a**,**c** and **e)** represent 1 μm, while (**b**,**d**,**f)** and insets represent 200 nm. (**g–i**) Surface morphology analysis, as indicated by black arrows. The dashed line in panel **i** represents pLFAO analysis (Fig. 2n). (**j**) Dynamic light scattering of fractions 18 of iUn, iFib, and iLFAO samples. **represents p < 0.05.

We have now established that LFAOs propagate as discrete units towards morphologically distinct fibrils (Figs 3 and 4). We have also understood that smaller units of sLFAO fibrils could be re-generated by sonication (Fig. 5). More importantly, it appears that iLFAOs are intermediates that are larger than pLFAO seeds. It is important to characterize iLFAOs and see how they differ from fragments generated from Un and sFib fibrils as they may reveal insights into strain-specific propagation of Aβ assemblies. Therefore, the morphology and size of each sonicated and isolated sample (iUn, iFib and iLFAO) were evaluated using AFM, dynamic light scattering (DLS) and other biophysical analyses (Fig. 6). Sonication results in the generation of short, ~120 nm length fragments in all samples (Fig. 6a–f). Although similar in length, iLFAOs (Fig. 6e and f) were discreet and unclustered as compared to iUn (Fig. 6a,b) and iFib samples (Fig. 6c,d). The surface amplitude analysis of iLFAOs revealed a repeat morphology (Fig. 6i) consistent with the morphology observed for sLFAO aggregates (Fig. 4m), which was absent with iUn and iFib samples (Fig. 6g and h). The constitution of ‘repeat morphology’ consisting of individual LFAO units was further confirmed by the similarity between the individual peak heights within iLFAO and pLFAOs (Fig. 6i, dashed line). Furthermore, an average of 4–6 peaks were observed for iLFAOs corresponding to the association of 4–6 individual pLFAO units (Fig. 4n). Together, the data confirms that iLFAOs are 4–6 times larger than pLFAOs and appear to be formed by association of individual pLFAO units. Although similar in size, iLFAOs are morphologically distinct from iUn and iFibs containing unique, ‘repeat morphology’. Size estimation of the iLFAOs, iFibs and iUn by DLS revealed a diameter of ~38 nm for iLFAOs and iUn while ~ 45 nm for iFibs (Fig. 6j).

To further analyze the similarities and differences between pLFAOs and iLFAOs, both of these samples were analyzed using 8-anilino-1-naphthalenesulfonic acid (ANS). Previously we have shown that pLFAO 12mers binds ANS less strongly than the 12-24mers^14^. Our current data indicates that 12-24mers bind ANS to a greater degree than iLFAOs or sLFAOs, suggesting that the former contains more solvent-exposed hydrophobic surfaces than the latter aggregates, which indicates conformational compaction during aggregation (Supplementary Fig. 2a). In addition, far-UV circular dichroism spectroscopy revealed that pLFAO 12-24mers contain a lesser extent of β-sheet as compared to iLFAOs or sLFAOs, further supporting the possibility of compaction during aggregation (Supplementary Fig. 2b).

### Numerical simulations identify the propagons within sLFAO fibrils

**Figure 7:**
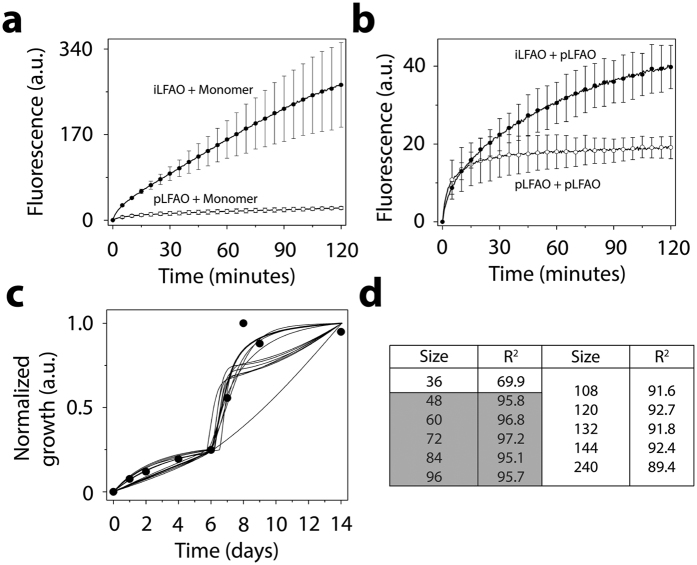
Identification of the propagating unit within sLFAO fibrils. (**a**) Aβ monomer (30 μM) seeded with 6 μM of either iLFAOs (●) or pLFAOs (○). (**b**) pLFAOs (10 μM) seeded with 2 μM of either iLFAOs (●) or pLFAOs (○). For both **a** and **b**, kinetic measurements were taken every 30 s beginning immediately after the addition of 10 μM ThT. (**c**) Numerical simulations of sLFAO aggregate formation (●, Fig. 3a), along with (**d**) R^2^ values for each respectively LFAO assembly (gateway) considered.

Having identified that iLFAOs are larger than pLFAOs, and that there may be stable intermediate during propagation, it becomes imperative to determine the size of the propagating units, which form the gateway for strain-specific propagation. The biphasic growth curve observed for sLFAO aggregation (Fig. 3a) showcases a possibility wherein an initial step may involve the generation of propagons prior to propagation. Therefore to understand this better, numerical simulations using ordinary differential equations (ODEs) was performed. Such an approach has provided important insights into the dynamics of Aβ aggregation^18^,^19^. To assist simulations, the ability of LFAOs (parent and isolated) to interact with and each other as well as monomers was evaluated by ThT fluorescence. Monomer elongation reactions involving the addition of monomeric Aβ42 on LFAOs showed that iLFAOs underwent elongation more effectively than pLFAOs (Fig. 7a). Similarly, oligomer elongation reactions involving the addition of pLFAO (12-24mers) on parent or isolated LFAOs also indicated that iLFAOs effectively interacted with pLFAOs as opposed to pLFAOs associating with each other (Fig. 7b). The rate constants for the simulations were included and initialized based on these observations and previously reported replication behavior of pLFAOs (12mers)^14^. The following criteria were considered in the simulations: *i*) since 12mers are more prone to replication than 12-24mers, it was assumed that during initial stages, association via monomer addition (formation of multiples of 12mers) predominates through sequential association of 12 monomers up to the formation of the propagon, and *ii*) the initial replication step involving the amplification of 12mers is not captured by ThT fluorescence as pLFAOs do not display high ThT binding^12^ and hence, was not considered in the model.

For simulations, the reactions considered were divided into two main types based on experimental observations (Fig. 7a,b) and other possible reactions: association of pLFAOs via monomer additions, monomer elongations and oligomer elongations. It was also assumed that while association via monomer addition predominates during the early stages, both the monomer and oligomer elongation reactions dominate during late stages of the reaction, with the latter to a more pronounced extent, although all reactions were considered. The detailed sets of equations considered are presented in the Experimental section. The simulations were performed by varying the propagon size from 24 to 240mers, and the simulation data were analyzed for the best fit with the experimental data to determine the propagon size. By doing so, the best fit was obtained for propagons 48-96mers (Fig. 7c,d), which corresponds to 4–8 pLFAO units.

## Discussion

**Figure 8:**
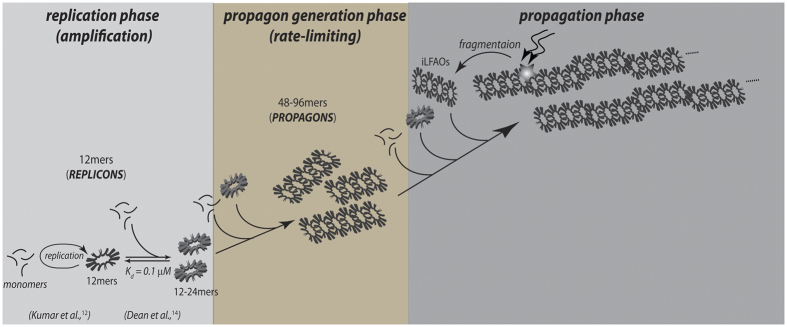
Mechanism of LFAO Propagation. At low concentrations (sub-μM) LFAOs are primarily 12mers (replicons) and undergo replication to form 12-24mers (amplification phase). It is important to bear in mind that LFAOs are not discrete oligomers but have some degree of heterogeneity and behave like a ‘cloud’. The schematic is only a cartoon representation of the oligomers and does not represent the actual structure. In a rate-limiting step, 12-24mers form LFAO propagons (48-96 mers), which undergo typical prion propagation to form large aggregates with unique morphology. iLFAO fragments can be generated from LFAO-seeded aggregates via sonication, which undergo both monomer and oligomer (pLFAO) elongation.

The data presented here reveal a prion-like, strain-specific propagation of a conformational phenotype of Aβ dodecamer called LFAOs. Although termed dodecamer, LFAOs have been shown to be rather disperse oligomers that more appropriately could be described as oligomeric ‘cloud’^12^. Recently we also identified that LFAOs dimerize at higher concentrations to form 12-24mers, which again indicates they are not discrete 24mers^14^. It is noteworthy that LFAOs are established to form along an off-fibril formation pathway and hence, compete with the on-pathway process^11^,^12^,^13^. In our previous reports, we have detailed the ability of LFAOs (12mers) to undergo a self-propagative replication mechanism in which LFAOs form quantitatively more of similar oligomers (12-24mers) upon interacting with monomer^11^,^12^,^13^. We have also established that replication is most efficient at low concentrations of LFAOs, where the oligomers are predominantly 12mers^14^. The observed inverse correlation between replication efficiency and LFAO concentration correlates with the concentration-dependent dimerization of the 12mer to 12-24mer and the latter’s diminished propensity to replicate^14^. When seeded with 12mer LFAOs (1 μM), the lag time of aggregation is marginally reduced (unpublished data), which could indicate that replication to form 12-24mers is a predominant reaction during the lag time. Upon achieving a threshold concentration of 12-24mers by replication, propagation towards higher order aggregates occurs, marginally reducing the lag time in the process. It is noteworthy that seeding of Aβ with high concentrations of LFAO seeds minimizes the emergence of on-pathway oligomers by altering the dynamics towards seeded aggregation. To understand the stability and seeding behavior during the propagation of sLFAO aggregates, sonication was adopted to shear away smaller fragments of fibrils and potential propagons. Although sonication is not the best technique to identify intermediates as it may induce inhomogeneous cleavage by brute force, the fragments of sLFAO still provide insights into their biophysical properties. However, we obtained rather homogenous oligomer fragments corresponding to 4–6 units of pLFAOs with a diameter of ~39 nm. A similar size range was also observed for Un and sFib (Fig. 6j), suggesting that the size of fragments produced by sonication do not preferentially cleave propagons from sLFAO aggregates. Therefore, iLFAOs cannot be considered as bona-fide propagons, although the size of iLFAOs (4–6 LFAO units) does agree to size obtained by molecular simulations (4–8 LFAO units). It is also noteworthy that propagons are a heterogeneous mixture of different sizes ranging from 4–8 LFAO units. The overall mechanism of LFAO-seeded aggregation appears to proceed in three distinct phases that involves sequential formation of replicons, propagons and fibrils (Fig. 8). These fibrils are morphologically and biophysically distinct from both those obtained from fibril-seeded and unseeded aggregates. From a mechanistic standpoint, our observation of three distinct phases of aggregation seems to parallel some of the recent reports implicating a secondary nucleation process in aggregation^20^,^21^. However, the results reported here are based on seeded aggregation and hence, a nucleation driven process is suppressed to a considerable extent, especially with high concentration of LFAO seeds.

Increasing evidence suggest that distinct phenotypes in AD pathology leading to varying progression rates, extent of neuronal death, and localization of pathology may arise due to the diversity among the aggregates of Aβ^22^,^23^. It has long been established by Tycko and co-workers that the variability in fibril growth conditions could manifest as microstructural differences and polymorphism within fibrils^7^. In addition, conformational plasticity among amyloid aggregates seems to form the basis for vastly differing phenotypes of AD^24^. Along the same lines, conformeric diversity and assembly differences among Aβ oligomers are observed to manifest in distinguishable pathological phenotypes in rapidly progressive AD^23^. These emerging reports along with those on prion-like propagation of Aβ^3^,^10^,^25^,^26^ bring forth the question of how oligomers of certain structure and assembly (conformeric strain) faithfully propagate their structure to homomorphic fibrils, and whether such differences manifest in phenotypic changes. The results presented here showcase an example where a dodecamer (LFAO) structure is propagated towards fibrils constituting a faithful reproduction of LFAO units within the fibril assembly, and provide insights into how strain-specific propagation could occur. More importantly, our results in TgCRND8 mice show that LFAO strains do not only propagate towards widespread amyloid deposition in brains, but also show selective pathological phenotype, which can be attributed to their structure. While fibril seeds show preferential deposition in cortex and hippocampus, LFAOs show remarkably higher propagation in cerebral vasculature leading to CAA phenotype. One of the main determinants in faithful propagation of LMW oligomer structure within fibrils is the relative structural stability of the oligomer, which facilitates the formation of HMW aggregates in either a templated or catalytic manner. So far, we know that LFAOs are formed along an off- pathway with relatively longer half-lives^13^, and at low concentrations (sub-μM) promote replication in a template-assisted manner^14^. Furthermore, LFAOs contain predominantly parallel β-sheets that are detected by the fibril-specific, conformational antibody, OC^13^,^14^. In addition, the surface hydrophobicity of LFAOs increases upon dimerization without changes in secondary structure^14^, suggestive of association induced structural organization. The propagation of LFAO-seeded fibrils reveals an initial replication step that serves as the prerequisite amplification step for propagation. Fibril fragmentation is mainly thought to be responsible for the observed exponential rates of propagation^27^. However, replication occurs free of fragmentation at low concentrations of seeding oligomers, providing insights into how threshold propagon concentrations could be achieved in physiological scales. Given the remarkable structural diversity observed among Aβ oligomers, it is likely that each of such ‘strains’ could propagate their structure and morphology in a biochemically faithful manner contributing to the observed phenotypic diversity in AD. This report has revealed molecular-level insights into one such strain providing important clues in such a mechanism.

## Methods

### Materials

Wild-type Aβ42 (Aβ) stocks synthesized from the Peptide Synthesis Facility at the Mayo Clinic (Rochester, MN) were used in this study. Monoclonal Ab5 antibody was generously gifted by Dr. Levites at the University of Florida (Gainesville, FL). The Superdex-75 HR 10/30 size exclusion chromatography (SEC) column was purchased from GE Life Sciences (Marlborough, MA). Gel electrophoresis and immunoblotting materials were purchased from either Bio-Rad Laboratories Inc. (Hercules, CA) or Thermo Scientific (Waltham, MA). Sodium salt of Lauric acid (C12:0) was procured from NuCheck Prep, Inc. (Elysian, MN). Super Signal West Pico Chemiluminescent Substrate was procured from Thermo Scientific (Waltham, MA). Trizma base, trizma hydrochloride, and SDS were purchased from Sigma-Aldrich (St. Louis, MO). All other chemicals were procured from Fisher Scientific (Waltham, MA).

### Preparation of Aβ monomers

Lyophilized stocks of synthetic Aβ were stored at −20 °C. Aβ monomer purification was performed by dissolving 0.8–1.5 mg of peptide in 0.5 mL of 10 mM NaOH and allowing the sample to stand at 25 °C for 15 min prior to fractionation on a Superdex-75 HR 10/30 SEC column on an AKTA FPLC system (GE Healthcare, Buckinghamshire). Monomer was purified at a flow rate of 0.5 mL/min and one-minute fractions were collected, and concentrations of Aβ fractions were determined by UV-Vis spectrometry on a Cary 50 spectrophotometer (Varian Inc.) using a molar extinction coefficient of 1450 cm^−1^ M^−1^ at 276 nm (expasy.org). Periodically, the peptide purity after SEC fractionation was confirmed by MALDI-ToF mass spectrometry, which revealed a monoisotopic molecular mass of 4515 Da. Aβ monomers were stored at 4 °C and used within 48 h of purification.

### Preparation of LFAOs and fibrils

LFAOs were purified as described previously^12^. Briefly, freshly purified Aβ monomer (50 μM) was incubated with 50 mM NaCl and 5 mM C12:0 fatty acid at 37 °C in quiescent conditions. After 48 h of incubation, the sample was subjected to centrifugation at 18,000 *g* for 20 min prior to SEC purification. Fractions corresponding to the peak near the void volume were collected. LFAOs were stored at 4 °C and used within 72 h of purification. Aβ fibrils were generated by incubating Aβ monomer (100–150 μM) in 150 mM NaCl with 0.01% NaN_3_ at 37 °C in quiescent conditions for 4–5 days. After incubation, the sample was subjected to centrifugation at 18,000 *g* for 20 min, and the pelleted fibrils were washed 3 X with 20 mM Tris (pH 8.0). Aβ fibrils were then resuspended in fresh 20 mM Tris-HCl (pH 8.0) at a final concentration of 50 μM. Aβ fibrils were kept at 4 °C and used within three weeks. Thioflavin-T (ThT) fluorescence and centrifugation was used to determine percentage conversion to Aβ fibrils, which was typically 80–90%.

### Seeding reactions

Reactions were initiated by seeding Aβ monomer (50 μM) with 10 μM of either LFAOs or fibrils. Aβ monomer in the absence of a seed was used as a control. All reactions contained 0.01% NaN_3_ and samples were kept at 25 °C in quiescent conditions for 14 days. Samples were then transferred to 4 °C for an additional 14 days of incubation. After a total of 28 days of incubation, samples were sonicated as described below.

### Thioflavin-T fluorescence

Data were collected daily by mixing 70 μL of ThT (10 μM) with a 5 μL aliquot of the sample. After a 1 min equilibration period, fluorescence kinetics was measured with excitation at 452 nm and emission at 482 nm over a 1 min period.

### Denaturing and non-denaturing PAGE with immunoblotting

Samples were diluted into 1X Laemmli loading buffer either with (denaturing) or without (non-denaturing) 1% SDS, and loaded onto either NuPAGE 4–12% Bis-Tris or 4–20% BioRad TGX gels without boiling. For SDS-PAGE, pre-stained MW markers (Novex Sharp Protein Standard, Life Technologies) were run in parallel for MW determination. Proteins were transferred to 0.2 μm nitrocellulose membrane (BioRad) and boiled for 1 min in a microwave oven in 1X PBS, followed by blocking for 1.5 h in 1X PBS containing 5% nonfat dry milk with 1% tween 20. Blots were then probed overnight at 4 °C with a 1:6000 dilution of Ab5 monoclonal antibody, which detects amino acids 1–16 of Aβ. Blots were then incubated with a 1:6000 dilution of anti-mouse, horseradish peroxidase conjugated secondary antibody and developed with ECL reagent (Thermo Scientific).

### Sonication and isolation

The samples were sonicated using a Misonix (Farmingdale, NY) XL-2000 series unit with a 2.4 mm diameter microprobe. Aliquots of samples (650 μL) in 1.5 mL siliconized tubes were sonicated (on ice) for 7 cycles (5 s sonication/20 s rest) at 80% power. The microprobe was used and kept at a constant depth of 17 mm inside of the sample during sonication.

### Dynamic light scattering (DLS)

DLS was performed on a Zetasizer Nano S instrument (Malvern, Inc., Worcestershire, UK). Each sample measurement consisted of 100 runs of 5 s each with a pre equilibration time of 30 s. The average of 100 runs was used to determine the diameter (nm) using the volume (%) function.

### Circular dichroism (CD)

CD spectra were obtained in the far-UV region with a Jasco J-815 spectropolarimeter using a 0.1 cm path length quartz cuvette (Helma) and monitored in continuous scan mode (260–195 nm). Acquisition parameters were 50 nm/min with 8 s response time, 1 nm bandwidth, and 0.1 nm data pitch. Each spectra represents an average of 6 scans. Averaged spectra were smoothed using the savitzky-golay algorithm with a convolution width of 15 using the Jasco spectrum analysis program.

### Atomic force microscopy (AFM)

Mica was cleaved using a razor blade and taped to a magnetic sample holder. The mica stub was then covered with 150 μL of 3-aminopropyltriethoxysilane solution (500 μL of 3-aminopropyltriethoxysilane in 50 mL of 1 mM acetic acid) for 20 min. The solution was decanted, and the mica was rinsed 3 times with 150 μL of deionized water. After rinsing, the mica stub was dried with compressed N_2_ gas and stored in a desiccator for 1 h. Next, 150 μL of 0.9 μM Aβ sample in 20 mM Tris buffer (pH 8.0) was added to the mica and allowed to adsorb for 30 min. The sample was then decanted, and the mica stub was rinsed three times with 150 μL of deionized water. Finally, the mica stub was dried with compressed N_2_ gas and stored in a desiccator until imaging. The surface topography of each sample was explored by imaging the peptide via a Dimension Icon atomic force microscope (Bruker). Measurements were taken under ambient environmental conditions at a constant scan rate of 0.5 Hz in tapping mode using ScanAsyst Air silicon tip on nitride lever (cantilever length, 115 mm; nominal force constant, 0.4 N/m; and resonance frequency, 70 kHz) (Bruker). The scan size ranged from 5 μm × 5 μm to 1 μm × 1 μm and the resolution was kept constant at 512 × 512 data points. AFM scanning was performed using Nanoscope 5.30r2 software and the images were analyzed using Nanoscope Analysis 1.50 image analysis software (Bruker). Multiple areas were imaged for each sample, and whereas height, phase, and amplitude data were collected simultaneously, amplitude images were used most often in the text to discuss morphology variation between samples.

### Molecular simulations

Ordinary differential equations (ODE) based numerical simulation was performed to simulate propagation reactions. The following set of equations were considered, where *O*_*12*_ is pLFAO and *O*_*n*_ is multiples of pLFAOs, *M* is monomer and *F* is LFAO strain-specific fibril:

#### Association via monomer addition


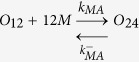



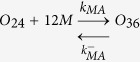



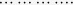



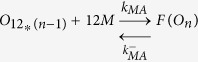


#### Monomer elongation


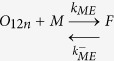


#### Oligomer elongation


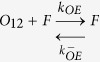



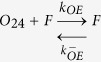



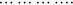



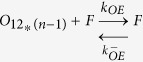


The reaction fluxes for these can be described in the following rate equations.


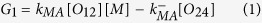



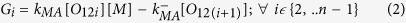



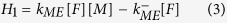



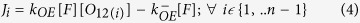


Based on these equations ODEs were formulated as a function of time for each species.


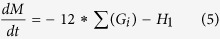



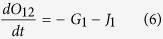



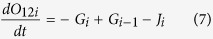


The differential equation of the gateway can be written as


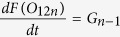


The calculated ODE was solved using Matlab ODE solver. First, the simulations were done for forward rate constants from 10^−5^, … 10^+5^ and backward rate constants from 10^−5^, … 10^+5^. For each combination the least mean square error between experimental and simulated data were calculated. The few best least error simulations are checked manually to compere the agreement between experiment and simulation data. The rate constants were sometimes manually tuned to better the fit with the experimental data.

### Animal work

All animal procedures were approved by the Institutional Animal Care and Use Committee (IACUC) at the University of Florida. All methods and procedures were performed in accordance to the guidelines and regulations. APP transgenic TgCRND8^16^ were bred in-house. Neonatal ICV injections with 1–4 mL of Aβ monomers, fibrils or LFAO (10 μM) were performed similarly to described before^15^.

#### Aβ ELISA

Mouse brains were sagitally dissected and left hemisphere was used for protein extraction using a sequential extraction protocol of RIPA buffer, 2% SDS and 70% formic acid as described previously^28^. All ELISA results were analyzed using SoftMax Pro software (Molecular Device).

#### Immunohistochemical imaging and image processing

Following tissue harvesting, the right hemisphere was formalin fixed, paraffin embedded and used for immunohistochemistry. Immunohistochemical staining was done using anti-pan- Aβ mAb5Biotin antibody (1:1500^28^), (biotinilated antibody was chosen in order to eliminate mouse IgG background staining interference). Immunohistochemically stained sections were captured using the Scanscope XT image scanner (Aperio) and analyzed using ImageScope program. Aβ plaque burden was calculated using the Positive Pixel Count program (Aperio). At least three sections per sample, 30 μm apart, were averaged by a blinded observer to calculate plaque burden. For evaluation CAA, sections stained with anti-pan- Aβ mAb5Biotin antibody were evaluated by a blind observer and blood vessels scored for presence of positive staining. 0 – no staining, 1–25% stained, 2–50% of the vessel has amyloid, 3 – entire vessel is stained. Vessels that received a score of 2 or 3 were counted in three sections per sample.

## Additional Information

**How to cite this article**: Dean, D. N. *et al*. Strain-specific Fibril Propagation by an Aβ Dodecamer. *Sci. Rep.* **7**, 40787; doi: 10.1038/srep40787 (2017).

**Publisher's note:** Springer Nature remains neutral with regard to jurisdictional claims in published maps and institutional affiliations.

## Supplementary Material

Supplementary Information
