# Supplementary material for: Strain-specific Fibril Propagation by an Aβ Dodecamer
Source: Sci Rep. 2017 Jan 18;7:40787. doi: 10.1038/srep40787 (PMC5241678; doi:10.1038/srep40787)
Supplement: Supplementary Information [file srep40787-s1.pdf]

Strain-specific Fibril Propagation by an Aβ Dodecamer

Dexter N. Dean[1], Pradipta K. Das[2], Pratip Rana[3], Franklin Burg[4], Yona Levites[4], Sarah E. Morgan[2], Preetam Ghosh[3], and Vijayaraghavan Rangachari[1*]

[1]Department of Chemistry and Biochemistry, University of Southern Mississippi, Hattiesburg, MS 39406

[2]School of Polymers and High Performance Materials, University of Southern Mississippi, Hattiesburg, MS 39406

[3]Department of Computer Science, Virginia Commonwealth University, Richmond, VA 23284

[4]Center for Translational Research in Neurodegenerative Disease, University of Florida, Gainesville, FL 32611

SUPPLEMENTAL INFORMATION

SUPPLEMENTARY FIGURE 1

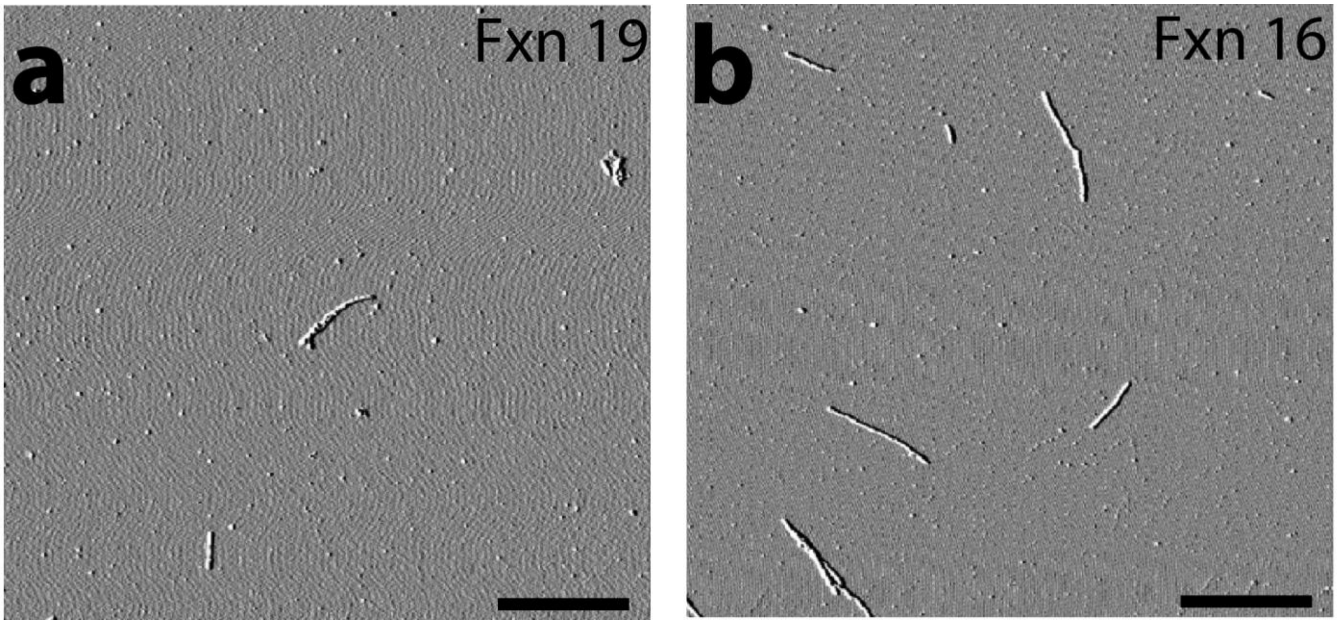

Supplementary Figure 1: AFM of sLFAO aggregate peaks from SEC. Representative AFM images of fractions 16 (**a**) and 19 (**b**) from sLFAO at 7 days of incubation. Scale bar represents 1 μm.

SUPPLEMENTARY FIGURE 2

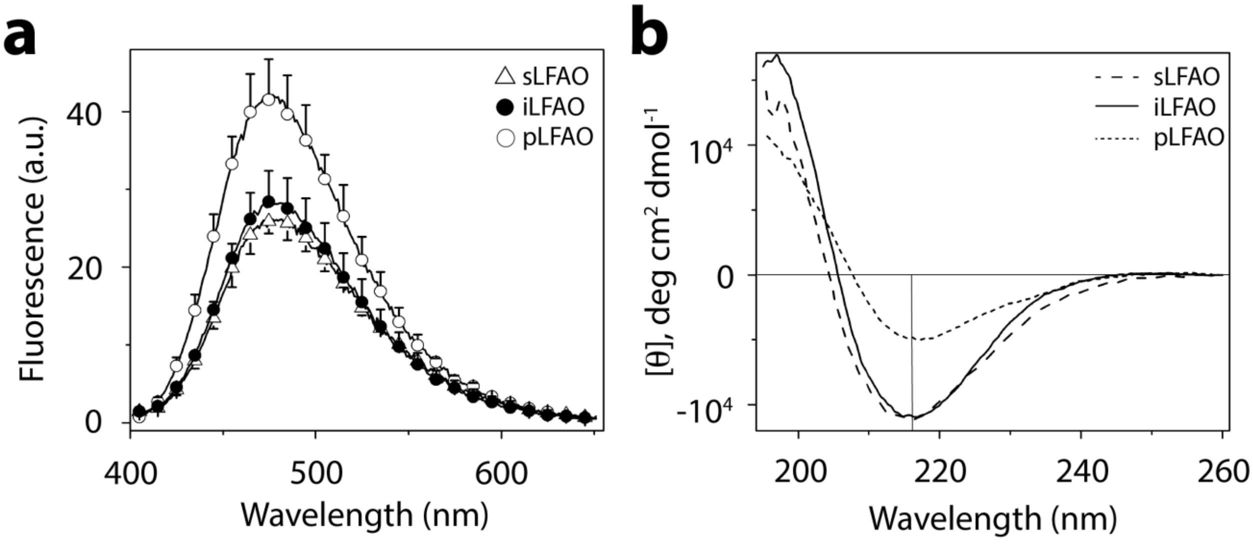

Supplementary Figure 2: Biophysical comparison of pLFAOs, iLFAOs and sLFAOs.

(**a**) ANS (100 μM) fluorescence scans of 10 μM sLFAO (△), iLFAO (●), and pLFAO (○), respectively. Samples were equilibrated 1 m before measuring fluorescence using an excitation of 388 nm and scanning emission between 405 and 650 nm. (**b**) Circular dichroism spectra for 10 μM sLFAO (dashed), iLFAO (solid), and pLFAO (dotted), suggesting a β-sheet secondary structure (minima at 217 nm).
